# COVID-19 public health and social measures: a comprehensive picture of six Asian countries

**DOI:** 10.1136/bmjgh-2022-009863

**Published:** 2022-11-07

**Authors:** Chuan De Foo, Monica Verma, See Mieng Tan, Victoria Haldane, Katherine Ann Reyes, Fernando Garcia, Carmelita Canila, Joseph Orano, Alfredo Jose Ballesteros, Tiara Marthias, Yodi Mahendradhata, Titiporn Tuangratananon, Nattadhanai Rajatanavin, Warapon Poungkantha, Tran Mai Oanh, Ong The Due, Nima Asgari-Jirhandeh, Viroj Tangcharoensathien, Helena Legido-Quigley

**Affiliations:** 1 Saw Swee Hock School of Public Health, National University of Singapore and National University Health System, Singapore; 2 University of Toronto Dalla Lana School of Public Health, Toronto, Ontario, Canada; 3 School of Public Health, Pamantasan ng Lungsod ng Maynila, Manila, Philippines; 4 Alliance for Improving Health Outcomes, Quezon, Philippines; 5 College of Public Health, University of the Philippines, Manila, Philippines; 6 Independent Researcher, Quezon, Philippines; 7 Department of Public Health, Gadjah Mada University Faculty of Medicine Public Health and Nursing, Yogyakarta, Indonesia; 8 The University of Melbourne Nossal Institute for Global Health, Melbourne, Victoria, Australia; 9 International Health Policy Program, Ministry of Public Health, Nonthaburi, Thailand; 10 Health Strategy and Policy Institute, Ministry of Health, Hanoi, Viet Nam; 11 Asia-Pacific Observatory on Health Systems and Policies, World Health Organization, New Delhi, India

**Keywords:** COVID-19, Health policy, Health systems, Public Health

## Abstract

The COVID-19 pandemic will not be the last of its kind. As the world charts a way towards an equitable and resilient recovery, Public Health and Social Measures (PHSMs) that were implemented since the beginning of the pandemic need to be made a permanent feature of health systems that can be activated and readily deployed to tackle sudden surges in infections going forward. Although PHSMs aim to blunt the spread of the virus, and in turn protect lives and preserve health system capacity, there are also unintended consequences attributed to them. Importantly, the interactions between PHSMs and their accompanying key indicators that influence the strength and duration of PHSMs are elements that require in-depth exploration. This research employs case studies from six Asian countries, namely Indonesia, Singapore, South Korea, Thailand, the Philippines and Vietnam, to paint a comprehensive picture of PHSMs that protect the lives and livelihoods of populations. Nine typologies of PHSMs that emerged are as follows: (1) physical distancing, (2) border controls, (3) personal protective equipment requirements, (4) transmission monitoring, (5) surge health infrastructure capacity, (6) surge medical supplies, (7) surge human resources, (8) vaccine availability and roll-out and (9) social and economic support measures. The key indicators that influence the strength and duration of PHSMs are as follows: (1) size of community transmission, (2) number of severe cases and mortality, (3) health system capacity, (4) vaccine coverage, (5) fiscal space and (6) technology. Interactions between PHSMs can be synergistic or inhibiting, depending on various contextual factors. Fundamentally, PHSMs do not operate in silos, and a suite of PHSMs that are complementary is required to ensure that lives and livelihoods are safeguarded with an equity lens. For that to be achieved, strong governance structures and community engagement are also required at all levels of the health system.

**WHAT IS ALREADY KNOWN ON THIS TOPIC**

- The COVID-19 pandemic had driven countries to implement an array of Public Health and Social Measures (PHSMs), which aim to mitigate the spread of the virus and minimise economic fallout. Most modelling studies highlight the effectiveness of one PHSM as a standalone measure but do not emphasise the interplay between PHSMs and the key indicators that determine their overall effectiveness.

**WHAT THIS STUDY ADDS**

- We have derived nine typologies of PHSMs and their accompanying six key indicators. The dynamic interplay between PHSMs and key indicators dictates the strength and duration of PHSMs.

**HOW THIS STUDY MIGHT AFFECT RESEARCH, PRACTICE OR POLICY**

- We found that for PHSMs be effective, national responses will need to place the most vulnerable first by centring PHSMs around an equity lens with strong governance and community engagement as key tenets at all levels of the health system.
- As the world moves towards living with the virus endemically, coupled with waning vaccine immunities, newer variant strains and the reopening of economies, health systems need to be made resilient and nimble to readily redeploy PHSMs. Such policy actions will help curb any spike in infections in order to protect the lives and livelihoods of populations.

## Introduction

 COVID-19, caused by SARS-CoV-2, which first emerged in 2019, has thrown the world into turmoil. At the end of May 2022, there were approximately 6.2 million deaths and 520 million cases globally.^1^ However, WHO estimated 14.9 million excess deaths (directly and indirectly associated with COVID-19) in 2020 and 2021 globally.^2^ With the emergence of more transmissible variants such as Delta and Omicron and COVID-19 vaccine inequities across countries, the virus is forecasted to stay with newer strains expected to surface.^3^ As seen in most countries since the start of the pandemic, Public Health and Social Measures (PHSMs) were implemented at varying intensities and durations to interrupt community transmission and mitigate the unintended consequences that PHSMs brought to the population.^4 5^

PHSMs had become commonplace in peoples’ lives since 2020, with countries that took decisive action suffering from fewer cases and mortalities and a more contained economic decline.^6^ As the crisis persists, certain countries have tapered off PHSMs and resumed the economy, despite the emergence of more transmissible strains.^7^ In fact, countries that lifted PHSMs prematurely had experienced a swell in caseloads, overwhelming of health systems and unwarranted mortalities, while others have reinstated targeted lockdowns that hinder economic recovery.^8 9^ Notably, vulnerable groups suffer from greater mortality and morbidity. Coupled with the absence of adequate social support, the pandemic has widened social inequities and reversed the course of many social and health indicators.^10^

However, the goals of PHSMs remain to prevent infection, contain outbreaks and blunt peak epidemic size so that healthcare systems are not overwhelmed at every step of the pandemic trajectory.^11^ To achieve this, PHSMs need to be commensurate with the intensity of contagion transmission and PHSMs safely adapted to ensure feasibility, sustainability and congruence to the local context.^12^ In fact, simulation models have investigated the efficacy of some PHSMs over others, but more importantly, no standalone PHSM can function effectively, but a comprehensive set is needed to curb local transmission.^13^ Notably, health systems that are resilient and can effectively and equitably implement PHSMs are postulated to be better positioned to protect the health and livelihoods of the population.^14 15^

In the face of newly emerging strains, breakthrough infections and reinfections among third dose vaccinated individuals, waning of vaccine immunity and pressure to resume the economy, prospects of living with SARS-CoV-2 endemically will necessitate agile adaptations of PHSMs with concurrent strengthening of health systems.^16^ To our knowledge, however, there is a paucity of data on the interactions among different types of PHSMs and between PHSMs and their key indicators that influence the strength and duration of their deployment. Therefore, this study aims to address this gap by analysing PHSMs implemented in six Asian countries through an in-depth case study analysis and provide insights into their interactions with each other and how key indicators directly impact the stringency and effectiveness of PHSMs. This study also contributes to an evidence-informed framework related to the strategic application of PHSMs.

## METHODS

### Country selection criteria

This study focuses on six countries in Asia that introduced different levels of PHSMs at different stretches of the pandemic. Although this study has a Asian regional focus, the selected countries differed in terms of income status, level of devolution and centralisation of governance, population density, mortality rates from COVID-19, health systems attributes, Universal Health Coverage Service Coverage Index and Global Health Security Index which give us a comprehensive picture of how PHSMs evolved and impacted national responses in a variety of six Asian countries. Table 1 provides a backdrop of country profiles, including key country indicators.

**Table 1:** Summary of key characteristics of six Asian countries

Country	Indonesia	The Philippines	Thailand	Vietnam	Singapore	South Korea
Income classification*	Lower middle	Lower middle	Upper middle	Lower middle	High	High
Life expectancy in males (years)†	69.6	67.3	73.5	71.3	81.4	80.3
Life expectancy in females (years)†	74.0	75.5	80.9	79.5	85.7	86.3
GNP per capita PPP in 2020 (current international $)‡	11 750	9040	17 710	8150	86 480	45 620
UHC service coverage Index in 2019§	59	55	83	70	86	87
Global Health Security Index for 2021¶	50.4	45.7	68.2	42.9	57.4	65.4
Total land area (km†) in 2018**	1 916 862	300 000	513 120	331 230	719	100 370
Population density (people per km^2^ of land area) in 2020††	146	368	137	314	8019	531
Cumulative confirmed deaths per million from COVID-19 by 20 January 2022‡‡	521.78	478.65	314.32	369.43	154.94	126.71
Proportion of population fully vaccinated by 1 January 2022§§ ¶¶	41	60	66	70	85	83

* Data taken from https://datatopics.worldbank.org/world-development-indicators/the-world-by-income-and-region.html#:~:text=The%20World%20Bank%20classifies%20economies,%2Dmiddle%2C%20and%20high%20income

† Data taken from https://www.worlddata.info/life-expectancy.php

‡ Data taken from https://data.worldbank.org/indicator/NY.GNP.PCAP.PP.CD

§ Data taken from https://data.worldbank.org/indicator/SH.UHC.SRVS.CV.XD

¶ Data taken from https://www.ghsindex.org/wp-content/uploads/2021/12/2021_GHSindexFullReport_Final.pdf

** Data taken from https://data.worldbank.org/indicator/AG.SRF.TOTL.K2

†† Data taken from https://data.worldbank.org/indicator/EN.POP.DNST

‡‡ DData taken from https://ourworldindata.org/covid-deaths

§§ DData taken from https://ourworldindata.org/covid-vaccinations

¶¶ Data for only the Philippines taken on 2 January 2022 from https://reliefweb.int/sites/reliefweb.int/files/resources/WHO PHL Sitrep 92_COVID-19 3 Jan 2022.pdf

GNPGross National ProductPPPPurchasing Power Parity UHCUniversal Health Coverage

### Literature search and case studies

The team of researchers from the six countries performed desk reviews of literatures relevant to PHSMs applied by their home countries. To that end, we used the literature review methodology set by Webster and Watson by harnessing scientific online data bases such as PubMed, EMBASE and Scopus as a starting point.^17^ Keywords during the initial search included ‘national responses to COVID-19’, ‘Public Health Measures’, ‘Social Support Measures’ and names of individual countries. We also performed a deeper dive into each PHSMs by repeating certain searches with the relevant PHSMs that we have identified after the initial thematic analysis. After the relevant articles have been sieved out, we selectively performed a backwards review of the pertinent citations from the articles to help address any missing data which we thought were applicable to our area of research.^17^ We also performed searches for national health accounts, government reports and policy executive summaries on the topic through digital government domains, mainly from ministries of health websites. To plug any remaining gaps, we performed a search of university databases where permitted, the websites of international development organisations and traditional and non-traditional media outlets (primarily through Google search). This provided us with a relatively complete census of the relevant literature made publicly available on PHSMs in the six countries. Data extracted in the local languages and English were included. Snapshots of the key PHSMs implemented from 2020 to 2022 are summarised in the results section and detailed in tables2^10^.

**Table 2:** Snapshot of physical distancing measures implemented in six Asian countries

	Indonesia	Singapore	South Korea	Thailand	The Philippines	Vietnam
Work from home (WFH) arrangements	WFH in Indonesia is part of the community mobility restriction policy, which at the beginning of the pandemic applies for all affected areas. At end of 2020, WFH implementation depends on severity (daily cases, level of restriction policy imposed on the area) within the area, which could be either at the district or provincial level. At a more micro level, in areas where low case load is recorded, offices would apply WFH should there is a local outbreak among the employees.	Telecommuting was advised since early 2020 but turned mandatory for nonessential workers when Circuit Breaker commenced. Snap WFH arrangements were enforced when huge spikes emerged in 2021. As Singapore learns to live with the virus, returning to workplaces are permitted although vaccination requirements and regular routine testing for workers.	WFH arrangements since the first wave was reinstated after spikes emerged from a rally that took place in August 2020. Going into 2021, South Korean businesses have built remote work infrastructure for the future, while relying on offline work when infections drop. WFH arrangements have persisted as infections surged in 2022 due to Omicron.	Advised voluntary WFH during first wave in 2020 and when the Omicron cases surged in July 2021.Adherence was low among non-salaried and informal sector workers.	An existing policy, Telecommuting Act enacted in 2019 was supplemented with new rules in March 2020 for fair remuneration and treatment for those who WFH. Employers are also required to offer adequate support for this transition. However, the huge informal economy had made WFH for a substantial proportion of the population challenging.	Since the start of the pandemic, WFH had been promoted based on epidemiological and hospital indicators, aligned with national Directives. As numbers remained high in 2021, most companies adopted hybrid work strategies. A ‘stay at work’ initiative where factory workers stay on premises to minimise contact with potential cases was trialled.
School closures	Schools were shuttered since March 2020 and remote learning was promoted but was faced with difficulties due to lack of internet access and learning devices. Schools reopened after 18 months when the numbers stabilised, but level of hybrid or remote learning was determined by local epidemiological severity.	Schools shut during Circuit Breaker and embraced full online learning. When cases dropped in mid-2020, staggered returns of graduating classes were permitted. Snap closures were enacted due to the Delta surge but have been reopened since mid-2021.	Since stoppage of physical lessons in early 2020, exemptions to user fees for educational websites and allowances for data usage was provided. Financial resources were given to needy students to procure learning devices. Full-time in-person classes resumed by the end 2021, although with high vaccination coverage in the general population but low in those aged 12–17.	Schools closed in 2020 and 2021 during the peaks. Sandbox Safety Zones were set up end 2021 giving local authorities the power to evaluate the situation and open schools accordingly. For severe zones, a minimum of 85% vaccination of school personnel and students were needed for reopening.	Schools had been shut since early 2020, with the cases remaining high and a slow vaccination campaign. More was needed to support blended learning, but the lack of internet access and devices stymied learning. Some schools reopened at the end of 2021, but all schools had to shut after an exponential growth in Omicron cases was experienced.	Telecommunications companies have agreed to waive internet charges for computers under an internet and computers campaign for children. However, the education gap is reported to have widened for those in poor and rural areas, with schools shuttered for the greater part of 2020 and 2021.
Restriction on public gatherings	Restrictions were based on the Implementation of Community Activity Restrictions (PPKM). A significant spike in cases in mid-2021 had resulted in the prohibition of gathering in public places and tightened limits on restaurant capacity and places of worship. Restrictions continue to vary based on location according to epidemiological severity and hospital capacities.	During the first wave, social gatherings were restricted and relaxed according to caseloads and health system capacity. Restrictions were re-tightened as the Delta wave threatened to overwhelm the health system, but the government is cautious to further relax measures as Omicron entered its borders.	The earlier part of 2021 saw stringent restrictions of public gatherings but as the social fatigue wore on, the government changed its stance to a ‘Distancing in Daily Life’ approach with a more nuanced approach. As Omicron swept through, limits to gathering numbers continue to be enforced and curfews for eateries persist while high-risk businesses such as nightclubs and bars remain open.	Public gatherings were limited since the beginning of the pandemic. In 2021, a zoning system was in place with dark red, red, orange and yellow zones based on size of epidemic. Limits on restaurant and mall capacities were also enforced. By the end of 2021, the system was revised to include a blue zone, which has minimum restrictions and are deemed safe to visit.	Mass gatherings were prohibited since 2020, based on graduated four-tiered system, with a higher-tiered categorisation for locations facing immense stress to their hospitals and large caseloads. Towards the end of 2021, gatherings in residences not belonging to the same household were permitted, while indoor capacities were limited to 50% of the full capacity with only the fully vaccinated allowed entry. Outdoor venues were also open at 70% capacity, without prejudice to vaccination status.	The levels of restrictions are based on three Directives, which will vary the level of restriction on public gatherings and curfews. Event capacities are also capped. Areas deemed as low risk can permit entertainment establishments to open while social events can accept only individuals who are vaccinated.
Lockdowns	Initially called Large Scale Social Restrictions was renamed PPKM which zones areas based on risks, epidemiology, surveillance and health services availability used to control stringency of measures, but a lockdown was never enacted.	Circuit Breaker (lockdown) measures kicked in during the peak of the first wave, mainly attributed to foreign worker dormitories and high community spread thereafter. Though lasting for 2 months, the spread was contained and there were no other lockdowns since.	No hard national lockdown but South Korea enforced stringent physical distancing measures in high incidence areas since the beginning of the pandemic. As the Delta wave caused hospital capacities to be overwhelmed, semi-lockdown measures kicked in for specific jurisdictions suffering from high infection numbers.	A nationwide lockdown was introduced in April 2020 with tight curfews in place to stem the first wave. As the Delta wave swept in July 2021, targeting lockdowns in Bangkok and provinces with high case numbers took effect.	The first lockdown took place in March 2020 in the Luzon island group, which included the Greater Manila Area, while individualised recommendations on the Community Quarantine levels differed based on the local epidemiological situation. Other cities and provinces soon followed suit by the end of March 2020. Highly restrictive community quarantines were instituted for specific cities and provinces depending on the estimated infection rates and healthcare system burden, such as the variant-driven surges in March 2021 and August 2021.	There were multiple waves that caused a nationwide lockdown, but flexibility was applied to each province and smaller administrative levels after the second wave.

**Table 3:** Snapshot of border control measures implemented in six Asian countries

	Indonesia	Singapore	South Korea	Thailand	The Philippines	Vietnam
Border closures	Started with a ban on travellers from China followed by a ban of travellers from high-risk countries and eventually to all countries. Into the second year, borders reopened slightly, with the need for all inbound travellers to have a vaccination certificate. Travel measures also applied for domestic travel with the need for a negative test result before departure. Domestic travel was suspended during peak season such as Ramadan to prevent surges.	In March 2020, all short-term visitors were barred from entering. In a bid to reopen to countries with close business and family ties, Reciprocal Green Lanes (RGLs) were opened towards the end of 2020 as the situation stabilised in selected countries. But as the Delta wave spread, RGLs cased. Vaccinated Travel Lanes (VTLs) started end of 2021 with over 20 countries but halted temporarily as Omicron caused surges locally and in other VTL countries.	Borders were shut since March 2020 to prevent entry of new infections. As vaccination rates increased, the country started its VTL with Singapore at the end of 2021 but had to temporarily suspend it due to growing numbers attributed to Omicron surfacing in both countries. South Korea continues to allow entry of residents, including long term pass holders but visa-free travel from most countries into South Korea remain suspended.	The country closed its borders in April 2020. However, many migrants had entered the country via relatively porous land borders. Domestic travel restriction during peaks of the pandemic. As the economy which relied heavily on tourism slumped, sandbox pilots such as the one in Phuket took off. Borders opened to fully vaccinated tourists in end 2021. But as the omicron circulated, the country banded flights from high-risk countries.	In late January 2020, borders started closing for travellers from Hubei, China, followed by the rest of the world as numbers skyrocketed and hospitals became overstretched. Border measures were relaxed slightly in 2021 but were tightened again after the Delta variant caused immense spikes in the community. Foreign travellers have been allowed entry to the country since the third quarter of 2020, although they must have a pre-existing visa. It was projected that borders and visa applications would be open to fully vaccinated foreign tourists in February 2022.	From March 2020 to July 2021, Vietnam closed its borders to international travellers while preparing its health system to absorb the spike in domestic numbers. In November 2021, Vietnam had sandbox pilots such as the Phu Quoc Island for inoculated travellers to visit without the need for quarantining. Since July 2021, Vietnam has been gradually reopening international routes to a number of countries in Asia, Europe and Australia.
Quarantine of inbound travellers	In early 2021, travellers needed to quarantine for 5 days and as the health system got overwhelmed, coupled with low vaccination rates and Delta surges, the period increased to 8 days. In early January 2022, 10-days quarantine period was implemented to prevent Omicron surges. This has been reduced to 3 days in March 2022. More recently in March, Bali, Bintan and Batam islands could receive international visitors without any quarantine period. Indonesian migrant workers, students and government personnel on official business will have their quarantine fees covered while foreign nationals bear full costs.	Those that left the country in March 2020 despite travel warnings will face the full cost of quarantine of 14 days. From September 2020, the duration was reduced to 7 days for travellers entering from low-risk countries. Delta strain outbreaks caused Singapore to increase quarantine periods to 21 days for travellers entering from high-risk countries regardless of RGLs. As numbers stabilised in 2021 and with higher vaccination rates, home quarantine was permitted for those who had conducive residential environments for quarantine. Those entering via VTLs need not quarantine but need proof of negative test results and vaccination status.	From the early phases of the pandemic, inbound travellers had to quarantine at designated facilities for 14 days and a GPS tracking function was used to ensure compliance. South Korea also permitted home quarantine in 2021 with daily monitoring using a self-diagnosis application. For travellers entering the country via VTLs, only proof of negative test results is needed before and on arrival.	All inbound travellers had to quarantine for 14 days at state facilities or select hotels during first half of 2021; shortening quarantine days gradually introduced. The government provided free testing for all nationals and non-nationals and full subsidised the cost of quarantine at government facilities. As the country opened up to low-risk countries in Q4 of 2021, full vaccinated travellers only need to wait in their hotels until negative RT-PCR on Day one.	In 2020, all travellers had to quarantine in appropriate facilities depending on symptom severity and test results. Returning residents need to quarantine at points of entry and after testing negative continue that remaining quarantine at home. In early 2022, all fully vaccinated travellers only need to show a negative test result before entering and self-monitoring for 7 days on arrival.	Vietnam cut its 14-day quarantine at centralised quarantine facilities for foreign travellers entering the country from 14 days to 7 days in August 2021 if they are fully vaccinated. The travellers will also be tested on the first and seventh day of quarantine. The Bluezone mobile application is required to be downloaded for the remaining 7 days of observation.

GPSglobal positioning system

**Table 4:** Snapshot of personal protective equipment requirements implemented in six Asian countries

	Indonesia	Singapore	South Korea	Thailand	The Philippines	Vietnam
Wearing of face masks for general population	Face masks became compulsory in April 2020, and during earlier stages of this requirement, the government urged the use of cloth masks in order to preserve surgical masks and N95 masks for health workers. A range of penalties both monetary and non-monetary was implemented and enforced by the police and military.	The population was advised to wear face masks only when unwell in January 2020. This advisory changed to mandatory face mask wearing from April 2020 with a fine of US$220 levied for non-compliance at the first instance and US$740 for repeated offences. Only under circumstances such as strenuous exercise or eating can face masks be removed. Safe Distancing Officers are employed by the government to enforce these rules.	Wearing of face masks was recommended on public transport in May 2020 but made mandatory in all public spaces in October 2020 after a sustained surge in infections was experienced. In July 2021, mask requirements were relaxed for fully vaccinated individuals but subsequently suspended after resurgence in infected caseloads.	There was early voluntary adoption of mask-wearing during the first wave and cloth masks were recommended to preserve surgical and N95 masks for health workers. In April 2021, wearing of face masks turned mandatory and a monetary penalty was enforced for non-compliance under the Communicable Diseases Act.	Mandatory mask mandate for all that are in public spaces was enacted in April 2020 but amidst rising infections and overwhelmed hospital systems in August 2020, compulsory face shield use was enforced as a further precaution against COVID-19. Towards the end of 2021, face shield requirements were lifted as vaccination rates increased and infections stabilised.	During the earlier stages of the pandemic, face masks were compulsory for those out in public and those who did not comply were slapped with steep fines. Cultural and environmental factors have led to high adherence rates throughout the pandemic as wearing of face masks were commonplace even before the pandemic.

**Table 5:** Snapshot of transmission monitoring measures implemented in six Asian countries

	Indonesia	Singapore	South Korea	Thailand	The Philippines	Vietnam
Contact tracing strategies	A contract tracing application, PeduliLindungi was developed in April 2020 to augment manual contact tracing efforts. As the restrictions started to ease in mid-2021, full digital contact tracing was adopted. WHO supports the training of contact tracers and the military and police force augmented civilian contact tracing manpower.	As cases from the first wave spiked, manual contact tracing was supplemented by TraceTogether (TT) mobile application and Safe Entry QR Code digital check-in systems. A physical TT token was issued to help those without a smartphone. These interventions have persisted since their implementation.	Contact tracing was done by checking medical facilities records, cellular geolocation, credit card histories and CCTV footage. The government subsequently introduced the COVID-19 Epidemiological Investigation Support System as a centralised data collection and multi-agency coordination platform, accelerating data request and approval procedures. In mid-2020, an electronic entry log, KI-Pass, was enforced.	Thailand had relied on village health workers to support official contact tracing teams. The initial strategy of using individual contact tracing was shifted to active case finding as numbers swelled during the second wave. However, individual case finding is still performed in areas with smaller clusters. Efforts were aided by a community-driven mobile application called ThaiChana. The use of the app became mandated for entry into all public spaces.	Contact tracing was to be conducted by Local Government Units (LGUs) overseen by the Department of the Interior and Local Government. Guidelines were set up since the nation’s brush with MERS. An active case finding strategy was adopted during the earlier stages of the pandemic with house-to-house symptom checking. StaySafe.ph had been launched as the official, centralised contact tracing system for COVID-19, although initiatives by different LGUs also produced parallel contact tracing modalities (eg, digital apps, paper-based forms) as cases continued to rise in 2020.	During its early stage, Vietnam used an aggressive contact tracing strategy with an epidemiological tracing of F0-F5 evaluation system. The use of contact tracing mobile applications was subsequently rolled out amid a backdrop of increasing caseloads, which sped up contact tracing efforts.
Surveillance strategies	A Public Health Operating Centre existed since 2017 and had a surveillance system in place that monitors infectious disease outbreaks via sentinel sites which have been linked to the country’s Early Warning Alert and Response System.	Sentinel surveillance was upheld in tertiary hospitals and primary care clinics since the beginning of the pandemic. A further routine rostered testing was initiated at all high-risk industries and cases reported to the Ministry of Health. As more strains entered the country, routine rostered testing (RRT) requirements were extended, and wastewater testing was deployed in residential estates.	After the experience with MERS-CoV, the Infectious Disease and Control Act incorporated aspects of surveillance into legislation. These provisions permitted a strong active surveillance approach with enabled health authorities to collect the same information as law enforcement in order to focus testing and quarantine on individuals who are more likely to be infected.	Surveillance had been an integral part of Thailand’s public health function before the pandemic, which performs sentinel surveillance in all 77 provinces. In the initial months of the pandemic, lab-based surveillance and data reporting was paper-based, but as the pandemic brought more infections throughout 2020, a national online laboratory and epidemiological database was formed to facilitate national surveillance.	A combination of events-based and sentinel surveillance is adopted by the Philippines. All private and public providers that identify confirmed or suspected cases are reported to COVID-19 coordinators that maintain and update the national COVID-19 information system. A COVID-19 tracker called COVID-KAYA was subsequently rolled out to electronically manage the heightened spread by consolidating data on all cases monitored, test results, health statuses from all accredited providers.	Vietnam used both active and passive surveillance strategies whereby prospective cases presenting at hospitals are tested and self-report through mobile applications. The country uses a suite of mobile applications including Bluezone and NCOVI to monitor the symptoms and track close contacts. The data is then synchronised to a centralised national database for surveillance purposes.
Testing strategies	At the start, testing of suspected cases was centralised. However, rigid criteria for PCR testing eligibility had limited testing numbers during the first few months of 2020. As the cases continued to overwhelm testing sites in 2021, RAT tests were permitted for rapid and early detection of infection. The cost of testing was covered by the government for suspected cases at public testing sites but not covered in private testing centres, but a price cap was instated in December 2020 for private testing which also varied based on jurisdiction.	At the initial phase of the pandemic, all suspected cases and close contacts were required to undergo PCR testing. In October 2020, RAT testing was explored and incorporated as part of existing RRTs. The costs of these tests were initially made free to the public, which can be taken at public and privately run testing centres. As the country moved towards living with the virus endemically, more social responsibility was levied on the population as they were given the prerogative to perform their own RATs, which were issued to every household by the government via the national postal service.	Emergency Use Authorisations were permitted for test kits as part of a multipronged national testing strategy. Initially, only PCR testing was used for persons suspected to be infectious, but as the numbers of infected individuals grew in 2021, self-testing was permitted. In the face of an Omicron wave, the protocol for prospective cases have shifted to those who tested positive with RAT do not need to take PCR before emerging from quarantine to save PCR tests for higher risk groups. Notably, the National Health Insurance Service covers the costs of tests for suspected and positive cases.	PCR testing served as the main gold standard from the beginning until the fourth wave in mid-2021 as testing bottlenecks become a massive challenge due to high demand from patients and high-risk contacts. The high demand was due to the need for a PCR result before hospital admission as home isolation was prohibited. The problem was relieved when RAT testing and home isolation became accepted. Importantly, the National Health Security Office covers the cost of testing for all nationals and the Ministry of Public Health agreed to cover the costs for non-nationals at both public and private testing centres.	Since May 2020, testing guidelines issued were stratified into four subgroups based on risk levels, with health workers or patients at risk of severe symptoms or with recent travel history prioritised for PCR testing. As testing capacity increased, PCR testing was offered to the general public. Testing services were covered by PhilHealth and a cap on co-payments at private testing sites were instated. The allowable conditions of use of RAT tests were expanded at the end of 2020 to include a wider spectrum of potential cases. A positive RAT test among close contacts is interpreted as a confirmed COVID-19 case, whereas asymptomatic close contacts who test negative with RAT test must have further confirmatory test (either through RT-PCR or a RAT).	In the first three waves, PCR tests were largely for people with symptoms, close contacts, inbound travellers and hospital patients. After the fourth wave when Delta emerged, testing strategy evolved to a large scale one with high testing frequency where more affordable rapid self-tests were used to detect and isolate source of infection, especially for asymptomatic cases while PCR tests were only used for confirmation purposes. The Health Insurance Fund covered the cost of testing for suspected cases and close contact of cases in both public and private testing centres.

MERS-CoVMiddle East Respiratory Syndrome CoronavirusRATrapid antigen test

**Table 6:** Snapshot of surging health infrastructure capacity measures implemented in six Asian countries

	Indonesia	Singapore	South Korea	Thailand	The Philippines	Vietnam
Surge capacity of treatment, isolation and quarantine facilities	To augment existing government hospitals, police and military hospitals become designated referral hospitals while other hospitals were urged to convert up to 40% of general wards to COVID-19 wards. An online platform that shows hospital bed occupancy was initiated. To rapidly increase capacity, Jakarta transformed its Asian Games Athlete’s Village into a COVID-19 makeshift hospital and quarantine facility. As traditional facilities get overwhelmed, ferries and ships were converted into floating isolation facilities in September 2021 to accommodate mild cases. All along, hotels were also used as self-isolation centres.	A 330-bed purpose-built facility which could increase to 500 beds was used to house patients since 2020. During the first wave, large venues such as convention halls were repurposed to community isolation facilities. Hotels also became facilities to isolate close contacts. The Delta wave brought an upswing in numbers needing isolation and community care facilities, renamed as community treatment facilities were set up to absorb cases that still require medical attention, particularly for the elderly and those with comorbidities. As hospitals reached capacity in the third quarter of 2021, the Home Recovery Programme was rolled out, augmented by telemedicine services. Private hospitals were also engaged throughout to boost overall health system capacity.	In early 2020, non-clinical amenities were converted into COVID-19 community facilities so that capacity for non-COVID-19 related services can continue. Concurrently, secondary and tertiary private hospitals were also engaged to add to the existing pool of dedicated COVID-19 hospitals while general hospitals were also transformed into respiratory split hospitals that could effectively segregate patients with respiratory symptoms with those that do not, limiting nosocomial transmission. As vaccination rates increased by the end of 2021 with more people suffering from milder symptoms or becoming asymptomatic, home isolation and recovery was promoted.	Hospitels (hotels repurposed as special hospitals) for cohort wards and keeping recovered patients were created at the outset. Military forts were also used as field hospitals for spare capacity. Exhibition halls and stadiums were also converted into field hospitals to expand overall capacity in 2020. As the fourth wave hit, home isolation was permitted in Bangkok, whereby doctors will communicate with patients remotely and food and medicines will be supplied at their doorsteps in a bid to relieve the strain placed on isolation facilities brought about by the Delta variant. Private hospitals were also engaged throughout the pandemic.	Selected government and volunteer private hospitals were designated as COVID-19 referral hospitals based on overall capacity and availability of human resources and appropriate infrastructure. In August 2020, One Hospital Command Centre was also launched as a coordinating centre for health facility referrals. Local Government Units identified and furbished provincial, city, municipal and barangay facilities that may be used as isolation facilities and to assist the Department of Tourism in identifying hotels and other similar establishments to be utilised as quarantine facilities. Simultaneously, government buildings, stadiums and event venues were repurposed for isolation usage. As the Delta variant caused an exponential surge, coupled with a sluggish vaccination drive, more health facilities were required in mid-2021 which came in the form of a request for more funds through the Health Facilities Enhancement Programme. Private hospitals were also engaged and reimbursed for providing service by PhilHealth.	To cater to the swelling cases from the first wave, large scale infrastructure including schools and stadiums, were converted into temporary COVID-19 hospitals. In mid-2021, as the country faced a huge wave derived from the Delta variant, more field hospitals were installed by military forces. Home isolation was also permitted for mild and asymptomatic cases with monitoring by local health commune centre staff. The government had been urging private hospitals to take on more COVID-19 patients since mid-2021 as public hospitals were overwhelmed due to the Delta wave.
Surge laboratory and testing capacities	At the beginning, all samples were sent to MOH appointed labs in Jakarta, resulting in slow processing of samples. As the pandemic progressed, testing became more decentralised. By mid-2021, Indonesia had almost doubled the total no of laboratories authorised for testing. As cases remained high through 2021, private laboratories were also engaged to provide drive-through and home-based testing.	Singapore had an existing national public health lab performing PCR testing during the first stage of the pandemic. In May 2020, the Testing Operations Centre which aggregated national demand and centrally manage allocation of capacity was formalised. By Mid-2020, as caseloads persisted, private GP clinics were engaged as Swab and Send Home clinics for more testing and surveillance in the community. To further meet demand, large scale quick test centres were set in October 2021 to performed supervised self-swabs while there was an increased emphasis on self-testing at home as all households were issued with RAT test kits.	Tests were initially centrally performed at the Korean Centre for Disease Control and subsequently scaled up to regional labs. Test kits were rapidly approved for use and sent to regional health centres at the start of 2020. Private sector labs were also partnered to augment government testing capacity. Concurrently, South Korea rolled out its innovative drive-through and walk-through testing to align with its aggressive national testing strategy.	Private labs were mobilised and certified by the Department of Medical Sciences under its ‘one province-one laboratory- 1-day reporting’ policy, ensuring sufficient testing capacities within all provinces. Thailand has a total of 500 government and private sector labs under its Laboratory SAR-CoV-2 Detection Network in 2022 after rapidly expanding capacity since the first pandemic wave.	The Research Institute for Tropical Medicine (RITM) was the lone laboratory conducting PCR tests in March 2020. To rapidly surge lab capacity, the Asian Development Bank and the Department of Health (DoH) convened Task Force T3, a private-public taskforce aimed at expansion of PCR capacities. It identifies the most urgent requirements for the set-up of PCR labs at priority hotspots. As waves continue to hit the Philippines, RITM and DoH continue to accredit more labs to join the National COVID-19 PCR Laboratory Network. By the end of 2021, licensed public and private COVID-19 testing laboratories numbered 287. Some private facilities implemented drive-through testing. The Philippine Red Cross also introduced saliva-based PCR testing in January 2021, whose results were retroactively included in official reports through a DOH policy in June 2021.	Since the first wave, Vietnam had been exponentially expanding its lab testing infrastructure, splitting the amenities between screening labs and confirmatory labs throughout all provinces by mid-2021. Many private laboratories that are qualified in terms of equipment and human resources have been authorised by the government as well. Mobile container labs that could provide up to 2000 tests per day were also deployed.

GPgeneral practitionerMOHMinistry of HealthRATrapid antigen test

**Table 7:** Snapshot of surging health supply measures implemented in six Asian countries

	Indonesia	Singapore	South Korea	Thailand	The Philippines	Vietnam
Domestic supply production and market initiatives	From the beginning, hoarding of medical supplies was prohibited. The Ministry of Health accelerated certification of services for production and distribution licences while offering a 1-day service for marking authorisation. To tackle medical oxygen shortages, the conversion of 90% of industrial oxygen into oxygen for hospitals was approved during the Delta wave. Additionally, plans to produce an antiviral pill to combat the Omicron wave was unveiled.	Singapore had promoted the setting up of domestic manufacturing capabilities such as ventilator production capabilities since mid-2020. Even in 2021, when demand for masks remained high, Singapore Technologies started producing medical-grade mask filters to resolve a critical vulnerability in its mask supply chain. Singapore had also inked deals with multiple pharmaceutical companies throughout the pandemic to ensure a wide portfolio of COVID-19 drugs.	KCDC directed private companies to produce diagnostic reagents and expedited approvals will be granted. In March 2020, the government intervened to purchase 80% of the mask supply from Korean manufacturers, fully banned exports, set a price limit on mask sales, and limited the no of masks sold weekly through retailers. People who hoard masks faced imprisonment and financial penalties.	Fabric and apparel companies also started manufacturing face masks by modifying existing production lines. Hand sanitisers were also reclassified as medical products to prevent unnecessary delays in their production. Manufacturers and distributors are required to inform the Internal Trade Department of the production cost, price, production volume and price labels to regulate prices of basic medical products.	Prior to the pandemic, the Philippines was not a local producer of medical-grade PPEs. As medical supplies dwindled, the government called for the manufacturing sector to shift production towards PPEs and ventilators. The coalition of Philippine Manufacturers of PPEs which is composed of electronics and garment manufacturers rapidly augmented existing supplies through government support since 2020. In August 2021, the Department of Health announced a price ceiling for PCR tests, cartridge-based testing and home service tests.	During the first outbreak, production lines that manufacture garments were converted to produce cloth masks instead. The government strictly prohibits arbitrary price increases, speculation or hoarding that affects the market for medical equipment for the prevention and control of COVID-19 across the country. Vietnam’s largest conglomerate also collaborated with external partners from other countries to manufacture ventilators for domestic use and eventually export.
National stockpile	No known national stockpile of medical supplies prior to the pandemic.	Singapore maintains a national stockpile of PPE, medical, critical medications and vaccines for up to 6 months even prior to the pandemic.	South Korea created a National Stockpile Plan for the management and distribution of medical countermeasures after an H1N1 influenza outbreak in 2009 to maintain stock of appropriate medical countermeasures, equipment and other supplies for outbreaks of these diseases, including testing supplies.	When the pandemic struck, Thailand had not maintained a national stockpile, the Kingdom was forced to look urgently for new external sources, explore domestic manufacturing alternatives and explore safe ways to reuse PPE. Efforts were made during the first months of 2020 to develop a national stockpile, but real-time digital inventory management was a major challenge, especially at the facility level.	No known national stockpile of relevant medical supplies prior to the pandemic. In February 2020, a bill was filed with the Senate seeking to establish a national supply-chain management office. This was to be achieved by amending the charter of an extant government-owned corporation, the Philippine International Trading Corporation, to institutionalise the stockpiling of critical materials in) response to or in preparation for national emergencies.	In September 2020, a national stockpile of medical supplies was recommended in preparation for future health emergencies.
Import and export regulations	All countries limited export of medical supplies while lifting import taxes and easing importation rules for import of medical supplies, especially during the initial stages of the pandemic, but loosened these measures as domestic and international production of medical equipment and resources picked up at the end of 2020 and beyond.					

KCDCKorean Centre for Disease ControlPPEpersonal protective equipment

**Table 8:** Snapshot of surging human resource capacity measures implemented in six Asian countries

	Indonesia	Singapore	South Korea	Thailand	The Philippines	Vietnam
Surge health work capacity	To augment the existing medical workforce, which was not optimal even before the pandemic, the government called for volunteers which included final year nursing students. However, health worker deaths affected overall workforce capacity, especially in 2021 when other more severe variants emerged. The military and police also deployed their health workers to aid in vaccination drives to accelerate inoculations.	When large clusters emerged in the migrant worker dormitories in 2020, the healthcare capacity was augmented with personnel from the Singapore Armed Forces and non-medical volunteers from regional health systems, to help contain the spread in the dormitories. In September 2020, the crew from Singapore Airlines were also tasked to assist healthcare workers at nursing homes. The Singapore Healthcare Corps, a platform to recruit volunteers both medical and non-medical was also set up in the early stages of the pandemic.	Integrated to the Central Disaster and Safety Countermeasures Headquarters, a total of 300 medical students were gathered and performed diagnostic swabs. The military was also called on to perform decontamination operations.	The government approved civil servant posts to aid in pandemic response by supplementing health professionals at medical facilities. Positions were also filled by contractual staff. Additionally, compensation was offered to the staff who contracted the virus. Thailand’s army of village health workers was also mobilised to for community engagement and medicine delivery services in the community.	At the beginning of the pandemic, the Department of Health led emergency hiring of health personnel and the newly hired would be deployed to facilities based on the order of priority: (A) designated referral hospitals, (B) temporary treatment and monitoring facilities, (C) designated diagnostic facilities, (D) public hospitals and (4) private hospitals designated to handle cases. Local Government Units (LGUs) also hired supplemental health workers for quarantine facilities, surveillance and other front-line work in relation to the pandemic. In April 2020, a memorandum allowed fresh medical graduates to be deputised physicians at a capacity limited to non-COVID-19 patients, even without a certificate of registration.	Since the first wave, retired medical staff were called on to augment the existing workforce. During the Delta surge, medical staff were reallocated from provinces with more controlled numbers to provinces with increased needs. The Central Youth Union was also called on to provide volunteers and teachers and students were also urged to register as volunteers in the hardest-hit areas. The military was also used to construct field hospitals, deliver basic necessities to households under isolation and aided in vaccination drives by facilitating logistic chains.
Surge personnel trained in FEPI/ Contact tracing	Several thousand graduates of health vocational schools have been trained since the beginning of the pandemic with WHO learning modules on contact tracing.	Since the first wave, contact tracers from the public service sectors were recruited and trained to augment existing manpower. Since mid-2021, there was a significant increase in the no of contact tracers trained due to large clusters. The additional contact tracing manpower came from various ministries and statutory boards with manpower further supplemented by external providers.	Its workforce of Epidemic Intelligence Service (EIS) officers was expanded by quickly training staff at approximately 250 local public health centres, hiring 300 private epidemiologists and leveraging staff at 11 non-governmental organisations that train and support EIS officers. Civil servants were also mobilised to take on the contact tracing roles.	Contact tracing was performed by over 1000 surveillance and rapid response teams with support from 1.1 million village health volunteers.	The Regional Epidemiological Surveillance Unit trains LGUs to contact trace and to use recording and reporting systems. In September 2020, the Technical Education and Skills Development Authority (TESDA) started offering free contact tracing training programmes and as numbers of infected cases spiked early 2021, TESDA urged LGUs to make avail its contact tracing programme.	In February 2020, the graduates from Vietnam’s CDC-supported Field Epidemiology Training Programme Short Course were deployed to provinces where the first clusters were located. To augment existing contact tracing teams, personnel from the military, public security and civil servants were activated to help with identification of close contacts.

CDCCenters for Disease Control and PreventionFEPIField Epidemiology Training Programme

**Table 9:** Snapshot of vaccine availability and roll-out implemented in six Asian countries

	Indonesia	Singapore	South Korea	Thailand	The Philippines	Vietnam
Vaccine supplies	Engaged the COVAX facility while procuring vaccines from multiple pharmaceutical companies before commencing vaccination drives. Domestic production began with state-own Biopharma to augment existing stocks while Indonesia receives donations from other countries.	Received the first shipment of vaccines at the end of 2020 and had continually been expanding its vaccine portfolio to meet increasing demands. Pharmaceutical companies have also inked agreements to set up plants in Singapore going forward to augment domestic and regional supplies.	In mid-2020, the government minted a deal between SK Bioscience and a pharmaceutical company to make core elements of the vaccine in the country. The government also made advanced purchase agreements with multiple vaccine manufacturers while continuing to formalise production agreements with other vaccine producing companies.	Thailand had domestically produced its own vaccine through a royal-owned drug manufacturing company but had to revise its vaccine acquisition plan in light of the Delta variant and lower vaccine efficacy for existing vaccines. Thailand joined COVAX in July 2021 in the face of supply constraints and high infection numbers.	Received its first batch of vaccines from COVAX in March 2021 as it continued to sign procurement deals with various drug companies to diversify its vaccine portfolio. The Asian Development Bank offered advance payments for vaccines and purchases of vaccine-related items. The World Bank had also made a contribution of 4.73 million doses in October 2021 to augment the supply chain.	In February 2021, a Resolution on the purchase of vaccines was ordained and carried out under the procurement mechanism in special cases under Article 26 of the Law on Bidding. The country also expedited vaccine use under its Emergency Use Authorisation protocols while supporting the formulation of home-grown vaccines for domestic production. Donations from other countries were also received.
Vaccine campaigns and requirements	Initiated vaccine campaign in January 2021, prioritising high-risk groups. However, vaccine drives slowed due to supply bottlenecks, weak cold chain and hesitancy. As more variants emerged, boosters were recommended in January 2022, with residents needing to pay for the extra shots. Vaccine mixing is not permitted.	VacciNationSG campaign was rolled out during the early months of 2021. As vaccination rates among the vulnerable increased, vaccines were made available in May 2021 to the general population at no cost. Booster shots were made available since September 2021 to the vulnerable and in October 2021, to the rest of the population. As vaccine efficacy wanes over time, coupled with more virulent strains, booster shots became mandatory to be considered fully vaccinated.	Vaccination differentiation measures kicked in a bid to further promote vaccination among the unvaccinated as more transmissible variants entered its borders. As the country launched its ‘Living with COVID campaign, it also launched a mobile app that displays vaccinated passports to facilitate vaccine differentiated measures. Further, the interval for booster shots was reduced from 4 to 5 months to 3 months in view of the Omicron incursion.	Thailand rolled out its mass vaccination drive in mid-2021. A booster dose was recommended end-2021 due to the spread of the Omicron variant and healthcare workers were also set for a fourth dose after 3 months from their third shot. To promote vaccination uptake, some village districts in Northern Thailand initiated incentives such as the chance to win a live cow every week for those who get inoculated.	National vaccination campaign (Resbakuna) was launched in March 2021. The phased implementation approach for the campaign was contingent on the projected availability of vaccine doses and it prioritised population groups according to risk of exposure (eg, front-line workers in health facilities and essential sectors) and risk of mortality (eg, poor population, senior citizens and adults with comorbidities). By December 2021, the waiting interval for boosters was shortened to 3 months to rein in the more transmissible Omicron variant. In early 2022, drive-through vaccination sites were set up. As hospitals continue to be overwhelmed in the face of rising Omicron infections, some policymakers expressed their support for severe restrictions for unvaccinated individuals (eg, prohibition from public transport and absolute stay-at-home orders except for accessing essential goods and services).	Initially, vaccines were prioritised to provinces with severe epidemic conditions and of economic significance. To maximise effectiveness of vaccines, mixing of vaccines was approved in July 2020. Vietnam also received vaccine doses from a few European countries through COVAX in January 2022. Multiple venues such as hospitals, district health and commune health centres served as vaccination sites. A unified mobile application that showcases vaccine certificates was also launched to facilitate vaccine differentiated measures.

**Table 10:** Snapshot of social and economic support measures implemented in six Asian countries

	Indonesia	Singapore	South Korea	Thailand	The Philippines	Vietnam
Support packages of general population	Unemployment benefits programme was introduced, this entail financial subsidies and training for those looking for new job or just lost their employment.	As the economy was hit by the pandemic, the SGUnited Skills Programme which aimed to provide job and traineeship opportunities to jobseekers was extended to March 2022 due to economic uncertainty. People who were quarantined were also provided with SGD 100 allowance during the peak of the Delta variant incursion in order to curb the spikes in September 2021 but this was removed subsequently.	Premiums for national insurance schemes were deferred while all COVID-19 treatments and tests were covered for South Korean nationals. In early 2020, the government issued Emergency Relief Disaster Payment to all households except those in higher income brackets.	A 6-month grace period was awarded to Social Health Insurance members whose employment was terminated or voluntarily resigned during the pandemic. After that, they were covered under the Universal Coverage Scheme. Utility bills were also subsidised for 4 months and e-vouchers were issued for a host of uses such as food and general goods.	A four-pillar socioeconomic strategy against COVID-19, which addresses the needs from all sectors of the population was implemented. For the general population, packages such as ringfencing US$23 million in Social Security Systemunemployment benefits and U$600 million for the Department of Agriculture programmes to ensure food security and agri-fishery support were implemented.	The government has issued various policies to support workers in the context of the COVID-19 pandemic, such as reduction of land rent, electricity bills, income tax, social insurance premium and a no of other fees and charges.Besides, 136 000 tonnes of rice from the national reserve was also issued to 29 provinces severely affected by the public health restrictions at the end of 2021, as measures persist due to the Omicron incursion.
Support packages for businesses	Tax incentives were given to selected businesses that supported the pandemic response such as companies producing medical products. Employees earning below a certain wage in these selected companies will be tax exempted.	Singapore Rediscover Vouchers (SRVs) were issued to all Singapore citizens aged 18 years and above to promote spending on local attractions to give local business demand for their services. The SRVs had its expiry date extended by 3 months in light of the pandemic’s persistence. The Job Support Scheme (JSS) was implemented in 2020 to support employers in retaining local employees. JSS payouts were intended to offset local employee wages to protect jobs. Since its enactment, the JSS has been extended multiple times till the end of 2021 due to continued fiscal uncertainty.	Self-employed and freelancers were provided with a job search promotion subsidy under the Employment Success Package and further monthly subsidy since April 2020. Business Closure Subsidies was offered to businesses in ‘hot zones’ to cushion the cost of closure. Remuneration for businesses continued into 2022 as strict public health measures persisted in the face of new variant incursions.	In 2020, a shopping subsidy package was disbursed to stimulate spending on goods and services to boost local demand for businesses. Soft loans were issued for small and medium enterprises in the tourism sector, and this was extended in 2021 due to the sustained slump in the tourism sector and overall economy due to border closures.	Support was provided to stakeholders in the food value chain such as the US$10 million stimulus package for the aquaculture sector. As the economy further weakened in 2021 due to sustained public health measures, the Corporate Recovery and Tax Incentives for Enterprises Act, or CREATE, reduced the income tax rate to 25% for big companies and 20% for smaller enterprises.	The government has issued three financial support packages, including US%2.6 billion in 2020 and US$1.1 billion in 2021 from government subsidy for employees, employers, and vulnerable people affected by the COVID-19 and US$1.3 billion from Unemployment Insurance Fund for workers unemployed due to COVID-19.Besides, reduction of 30% of corporate income tax in 2021 and employers and employees are suspended or need to only pay reduced premiums to the pension and survivorship fund for 6 months as businesses facing difficulties due to prolonged public health restrictions.
Support packages for vulnerable groups	Existing direct cash transfer programme such as Programme Keluarga Harapan catering to low-income households was expanded to support COVID-19 management since April 2020 and it had continued to be in place, but the quantum lowered in mid-2021 as the public health measures persisted in the face of a slow vaccination drives and high infection rates.	The Courage Fund provides financial support to low-income families whose member undergoes mandatory leave of absence/ stay home notice or quarantine order which impacts their livelihood. The COVID-19 Recovery Grant provides temporary financial support to workers in lower-income to middle-income households who are presently experiencing involuntary job loss, involuntary no-pay leave or income loss due to the economic impact of COVID-19. This was rolled out in January 2021 but has been extended due to uncertain job markets. Migrant workers need not pay for COVID-19 testing and treatment.	A proportion of all supplementary budgets approved during the pandemic was reserved for the vulnerable. This included emergency income supports and vouchers to the low-income brackets as infections remained high with the hospital system strained due to a high no of severe cases.	A cash transfer was offered for workers in the informal economy in the first few months of the pandemic. The government also provided eligible migrant workers with a one-off payment in mid-2020. COVID-19 testing, and treatment are also covered for migrant workers.	The Department of Social Welfare and Development received around Php 200 billion under Bayanihan 1 Act and US$117 million under the Bayanihan 2 Act for the implementation of social amelioration programmes. Households in areas under the most severe community quarantine level were entitled to receive a sum between US$ 100–US$150. Emergency subsidies were offered to recently returned overseas Filipino workers and unemployment or involuntary separation assistance for displaced workers or employees. Community pantries had also sprouted from a grassroots initiative that offers food items to the needy in the midst of extended lockdowns and a growing food security challenge.	Low-income households and workers whose employment was suspended received roughly US$45 per month for 3 months in 2020.Besides, pregnant women and people raising children under 6 years old received food support.

### Data analysis and presentation

The research team extracted key themes from the literature obtained from countries by performing an inductive thematic analysis whereby theoretical insights are generated from data,^18^ and thereafter drew comparisons across case studies which include all salient themes as typologies of PHSMs.^19^ Multiple rounds of iterative feedback between team members were performed to ensure the typologies correctly represented the themes that surfaced from the data to ensure reliability and accuracy. Any disagreement was resolved through discussions till a consensus was reached. Thematic saturation was reached when no new themes emerged.

## Results

The inductive thematic analysis applied by this study emerged nine PHSM typologies, which include (1) physical distancing, (2) border controls, (3) personal protective equipment (PPE) requirements, (4) transmission monitoring, (5) surge health infrastructure capacity, (6) surge medical supplies, (7) surge human resources, (8) vaccine availability and roll-out and (9) social and economic support measures. We analysed how these nine PHSMs interacted among themselves.

**Figure 1:**
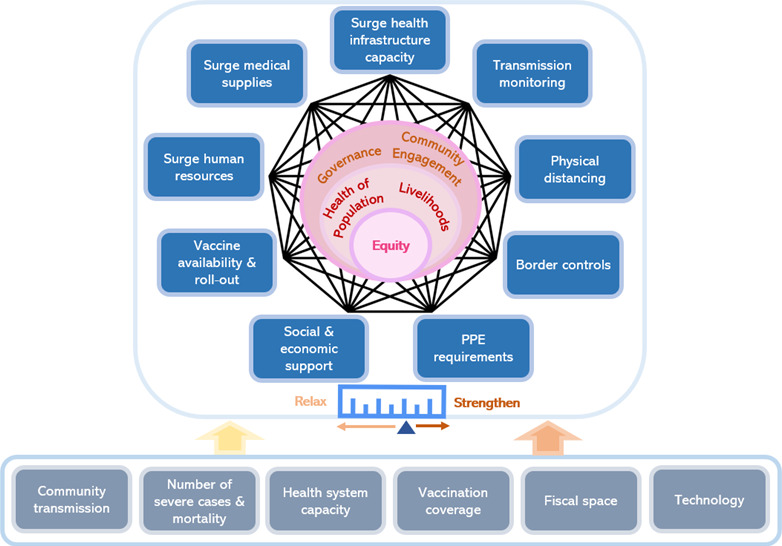
Framework of interactions among nine PHSMs and six key indicators that influence the scaling up and down of PHSMs. PHSMs, Public Health and Social Measures.

In addition, we identified six key indicators influencing the scaling up or down of PHSMs, including (1) size of community transmission, (2) number of severe cases and mortality, (3) health system capacity, (4) vaccine coverage, (5) fiscal space and (6) technology. Thereafter, we expand on the nine overarching PHSM typologies and analyse how the six key indicators shape the strengthening or relaxing of PHSMs, with reference to the synthesised framework in figure 1.

### Physical distancing

All countries reviewed implemented physical distancing measures to prevent viral transmission between people in close proximity. Common methods include school closures, work from home (WFH) arrangements or limitations applied to public gatherings. School closures vary in duration and intensity, with countries such as Indonesia, Thailand, the Philippines and Vietnam facing challenges of remote learning due to limited internet coverage and availability of learning devices. Similarly, despite digital challenges when shifting to WFH arrangements, all countries pushed for minimal office presence if offsite options were available. In addition, all countries reviewed introduced restrictions on public gatherings, most commonly implemented by controlling the size of group gatherings based on a tiered system, with lockdowns implemented only as a last resort. Singapore, for example, initiated its Circuit Breaker lockdown in response to massive outbreaks within foreign worker dormitories and to rapidly suppress community transmission. In larger countries, stringency of physical distancing differs based on epidemiological performance at sub-national levels, such as the Community Activity Restrictions (known as ‘PPKM’) in Indonesia, which tailored these restrictions based on local hospital capacity and caseloads.

### Border controls

The imposition of travel bans to stem the inflow of infections by countries was commonplace after the WHO declared a public health emergency of international concern. For inbound travellers, strict quarantine at designated facilities was required, with the need to monitor health status through various applications. South Korea, for example, mandated inbound travellers to quarantine in dedicated facilities while a Global Positioning System tracking function was used to monitor compliance. As the pandemic continued into its second year, countries relying heavily on tourism trialled calibrated reopening of their borders to the fully vaccinated through sandbox pilots such as the Phuket Sandbox in Thailand and Phu Quoc Island in Vietnam. By the end of 2021, certain countries had shifted from a static close all borders approach to a more dynamic one based on epidemiology in sending and receiving countries, vaccination statuses and to some extent, reciprocity. As vaccination coverage expanded, countries such as Singapore and South Korea commenced vaccinated travel lanes (VTLs), with vaccination and negative test requirements for inbound travellers. However, with the resurgence of more virulent strains such as Delta and Omicron, these countries retightened their borders iteratively.

### PPE requirements

Countries reviewed adjusted their requirements for mask-wearing based on the stage of the pandemic that they are in. For example, in the early stages of the pandemic in Singapore, mask-wearing was only for the unwell, but subsequently, everyone was required by law to wear one. Eventually, all countries implemented mandatory mask-wearing, with penalties imposed for non-compliance, such as in Indonesia, where police and military personnel meted out fines to those who flouted these mandates. However, amid increasing vaccine coverage, South Korea altered their masking requirements such that vaccinated people need not wear a mask outdoors by July 2021, but this was suspended amid a spike in numbers, while the Philippines had mandated everyone to wear both face shields and masks while in public. In some Western countries, mask-wearing had been a point of contention between policymakers and citizenry.^20^ However, this was not the case in countries reviewed due to cultural and environmental factors, such as in Vietnam, where wearing face masks was common due to air pollution in the cities.

### Transmission monitoring

Countries reviewed implemented extensive testing, surveillance and contact tracing strategies since the beginning of the pandemic to detect and isolate potential and confirmed cases quickly. PCR testing remains the gold standard for diagnosing COVID-19. Chiefly, PCR testing is coupled with other more efficient testing methods such as rapid antigen tests (RAT) when laboratory testing capacities became overwhelmed by high caseloads brought about by multiple epidemic waves and testing requirements.

Testing strategies are in tandem with contact tracing protocols which aim to identify cases to break transmission chains. Contact tracing teams were rapidly mobilised in all countries, embarking on a manual tracing process initially. As transmissions intensified, digital tools were used to accelerate the tracing process. In Singapore, the TraceTogether mobile application and Safe Entry QR code were created by the government technology office and adopted by the population to aid in contact tracing, while South Korea leveraged CCTV footage, cellular geolocation and credit card histories for the same purpose. However, uptake of such digital strategies faced challenges due to privacy concerns. For the Philippines, an additional hurdle was the parallel development of multiple information systems and digital platforms that were not immediately interoperable. Generally, passive surveillance was already in place for most countries with a system to detect underlying infectious diseases prior to COVID-19, and since 2020, was augmented with active and sentinel surveillance protocols through healthcare providers, routine rostered testing for workers or wastewater testing.

### Surge health infrastructure capacity

Health infrastructure such as laboratories, isolation facilities and negative pressure rooms in medical facilities are needed to identify and care for patients and suspected cases as well as parameterise them from the community to reduce transmission. Countries expanded laboratory testing capacities by setting up new laboratories or authorising private laboratories to provide testing services. In South Korea, novel testing protocols such as drive-through and walk-through testing were deployed in car park spaces. In Vietnam and Thailand, the distribution of testing laboratories proliferated across the country in order to reach all provinces. This was done by expediting accreditation of government and private laboratories, commonly under a national laboratory network system. Nonetheless, as more transmissible variants emerged, even this expanded capacity faced difficulties with the swelling demand, making RATs an acceptable means to triage patients for RT-PCR tests. As for quarantine and isolation facilities, countries partnered with the private sector and converted hotels and other large public facilities such as convention centres into quarantine or isolation facilities. Designated hospitals were also retrofitted to serve as respiratory split hospitals to segregate prospective COVID-19 patients from the general patient pool, such as those in South Korea. Initially, the focus was on the quarantine of contacts and isolation of cases in dedicated facilities resulting in a large emphasis on creating such facilities. However, countries such as Singapore and South Korea which had high population vaccination coverage had progressively moved towards a more home-based quarantine and isolation approach for patients with milder symptoms to be managed at home to preserve hospital capacity for more severe cases.

### Surging health supplies

At the early stages of the pandemic, countries preserved health supplies by easing import regulations while restricting the export of certain medical products, which were subsequently lifted after domestic production was increased through the use of innovative technologies and approaches. For example, garment factories in Thailand, the Philippines and Vietnam were retrofitted to manufacture PPEs while approvals for manufacturing or sale of certain health products were expedited. For instance, Thailand reclassified hand sanitisers as medical products to prevent production delays. Price controls and regulations to deter hoarding were implemented to ensure the population had access to available stocks. South Korea had initially limited weekly masks sales by retailers, while hoarders faced imprisonment and financial penalties. In addition, countries such as Singapore and South Korea maintained a national stockpile of medical supplies that could be rapidly deployed in times of health emergencies.

### Human resource capacity

Having a sufficient and healthy healthcare workforce is imperative to treating, isolating, testing and contact tracing during a pandemic. Across reviewed countries, healthcare workers were overburdened as case numbers increased while taking on heightened risks when managing COVID-19 patients, at times with uncertain access to PPE necessary to provide care safely. To mobilise more human resources, some countries activated their ‘reserves’ which predominantly came from former or retired healthcare workers, medically or non-medically trained volunteers, medical and paramedical students, or the military. In countries such as Vietnam, the military and public security forces were rapidly mobilised and trained to perform contact tracing. In Singapore, nursing students answered the call for volunteers by joining mobile serology teams, while Thailand successfully mobilised their pool of village health volunteers for public education activities and community surveillance.

### Vaccine availability and roll-out

Mass vaccination has been touted as a key exit strategy from this pandemic. However, vaccine supply chains must be secured, high-risk groups prioritised and distribution plus access within communities ensured through robust cold chains. Vaccine drives commenced at the beginning of 2021 for countries reviewed. As Singapore ramped up its vaccination drive, it unveiled its VacciNationSG Campaign to address misconceptions, debunk misinformation about vaccinations and promote uptake. Other strategies to promote vaccine uptake are allocation and affordability. In terms of allocation, all countries reviewed had prioritised high-risk groups such as the elderly and front-line workers for vaccination, with the costs of vaccination borne by the government. However, access to vaccines at the country level posed a hurdle for countries failing to secure sufficient advance purchase agreements with pharmaceutical companies. Donations from other countries and the COVAX facility had provided vaccine doses to countries such as the Philippines, Indonesia and Vietnam, although only for a marginal proportion of their population, while others such as Indonesia and Thailand produced their own. Most countries also implemented vaccine differentiated measures, such as Singapore, South Korea and Vietnam, which necessitate a digital vaccination certificate to be presented for entry into certain venues. As more transmissible variants circulated, countries began recommending booster doses, with Singapore only considering those with a booster dose as fully vaccinated.

### Social and economic support measures

All countries had provided support packages to the vulnerable groups and businesses, but the level and type of support for the general population varied. These included financial relief packages, skills upgrading to increase employability, measures that cover basic necessities, insurance premium waivers and utility subsidies. To revitalise the economy, Singapore issued vouchers to the general public to spend on local businesses, while Indonesia, Philippines and Vietnam provided tax incentives and reductions in corporate tax for selected businesses. Vulnerable groups such as low income and migrant workers were supported with cash transfers, unemployment protection and free COVID-19 treatment services. Some countries such as the Philippines saw the emergence of ground-up initiatives like food pantries, and Indonesia expanded its cash transfer initiative under Programme Keluarga Harapan for low-income households. Support packages were reportedly extended due to poor economic outlook and prolonging of PHSMs in the face of new waves of infection.

## Discussion

In each country, the nine typologies of PHSMs were introduced at different stages and intensities, adapted and influenced by key indicators in order to respond effectively as the pandemic unfolded. The types of PHSMs implemented, though similar in their fundamental policy structure and objectives, were enacted either at national or subnational levels depending on viral transmission, feasibility, fiscal capacity, health systems readiness and economic implications based on local context. The discussion section offers insight into the dynamic interplay among PHSMs and their effects on each other. This is followed by the key indicators that determine the strengthening or relaxing of PHSMs.

### The dynamic interplay among PHSMs

PHSMs cannot function alone and must be implemented strategically and synergistically to maximise the protection of the health of populations and livelihoods. Synergies across different PHSMs can achieve their intended outcomes and withstand unintended consequences. Three nested interactions that emerged from this study will be discussed.

First, transmission monitoring through testing and contact tracing must be followed by citizens’ compliance with quarantine and isolation to stem community transmission. Ringfencing cases and contacts require alignment with social and economic support measures so that these individuals possess the resources to remain temporarily segregated from the rest of the community.^21^ This may come in the form of food delivery services as seen performed by village health volunteers in Thailand and allowances or income replacement given to isolated individuals in Singapore. This is especially integral for informal economy workers or daily waged earners, who are disproportionately affected by these quarantine measures, making physical distancing measures difficult to adhere to.^22^ The need for such support is more evident in low-income and middle-income countries (LMICs), where a large proportion of their population belongs to informal sector and is impoverished.^23 24^ However, vulnerable groups also exist in upper-middle-income and high-income countries, which are disproportionately affected by COVID-19 who also need government support. This dilemma further adds to the complexities between the protection of the health of the population and livelihoods.

Second, a more complex interaction network between various PHSMs entails interactions between health worker capacity, health infrastructure, border controls, vaccination roll-out and testing requirements, to name a few. To elaborate, human resources are needed to expand health services provision capacities to respond to the pandemic and to maintain other essential routine services at health facilities, which can be accomplished by mobilising military personnel and volunteers to refurbish non-clinical facilities and erect field hospitals and vaccination centres. During vaccination roll-out, human resources are required for inoculation services and facilitation of the cold chain. Private sector providers are commonly engaged to augment government service personnel to conduct laboratory tests, treatment and quarantine services. In Thailand, public and private healthcare providers were contracted on the same terms, conditions and fees, which had to an extent, prevented patient denials by private facilities.^25 26^ To that end, hotels were also converted into isolation amenities for returning travellers and close contacts during the initial peaks. Closely related are sandbox pilots for travel, where vaccine campaigns were accelerated to reach a certain level of population coverage before reopening of borders, which was determined by the availability of vaccines, manpower capacity to vaccinate and relevant supporting infrastructure.

Third, the availability of PPE, medical supplies and human resources also need to complement one another to maximise their effectiveness. Specifically, in certain countries with initial supply shortfalls, mask coverage for the general population was low during the early stages of the pandemic. These mismatches between PHSM objectives and medically related supplies were alleviated after rapid market approvals were authorised and domestic manufacturing capacities bolstered. Similarly, adequate PPEs for healthcare workers must be preserved to safeguard essential workforces to render COVID-19 related services while maintaining other essential health services. The severe lack of PPEs had endangered the lives of health workers and, in some cases, precipitated into healthcare worker mortality, further straining the countries’ test, trace and test capabilities.^27 28^

The list of interlinkages, complementation and synergies between different PHSMs are non-exhaustive, as depicted in the framework by the different permutations represented by the intersecting black lines in figure 1. Fundamentally, the mantra that PHSMs do not operate in silos and one PHSM should not substitute but complement another must be amplified at all levels of the health system.^29^

### Key indicators influencing the dynamism of PHSMs

As the pandemic wore on, vaccines became more available and with the emergence of the more virulent strains in 2021, most countries adjusted their PHSM based on these emerging contexts as well as the impact of prolonged PHSMs on the economy. Policies had to balance between the health of the population and livelihoods. In general, the key indicators that govern the scale and duration of PHSMs are similar across the six countries. For example, Vietnam had used five explicit metrics to achieve this. Specifically, during the early stages of vaccination roll-out, a proportion of the population over the age of 50 fully inoculated was the most important indicator, as older persons were more predisposed to severe symptoms and overburden the health system, followed by the percentage of people over 18 who receive at least one vaccine dose. Next, the health system capacity indicator was predicated on the number of oxygen tanks at commune health stations to be used as a precaution for managing milder cases in the community along with the districts’ plan of deploying mobile health stations to provide care for hard-to-reach populations. Another indicator existed during the peak of demand for critical services, where the government earmarked ICU beds for the most severe COVID-19 cases based on an estimation of the percentage of total COVID-19 cases as potential demand. The number of community infections was also incorporated when calibrating the magnitude of PHSMs. Broadly, in all countries reviewed, parameters for decision-making towards scaling up PHSM include: community spread (indicator 1), number of severe cases and mortality (indicator 2) and health system capacity (indicator 3). For most countries, excluding Singapore, decisions were also made at the subnational level, and PHSMs tailored based on subnational key indicators.

n addition, a country’s fiscal capacity (indicator 4) and vaccination coverage (indicator 5) also act as important indicators (25). Countries that depend heavily on tourism, with large informal economies or insufficient budget reserves, might find themselves reopening their economy prematurely.^30^ Countries such as Thailand whose economy relies on tourism cautiously reopened borders at selected destinations, contingent on high vaccination coverage. Moreover, of the total government debt, 59.5% of GDP in 2022, 98.14% were internal borrows and were used to finance social and economic support and recovery from the pandemic.^31^ In 2022, vaccine policy changes were influenced by demand for booster doses in the light of waning vaccine immunity, breakthrough infections, new vaccines and existing fiscal space. When Omicron circulated towards the end of 2021, huge waves of infections threatened to overwhelm health systems. As a result, Thailand recommended a fourth dose to health workers, while Singapore mandated a booster dose to be considered fully vaccinated.^32 33^ However, despite high vaccination rates in countries such as Singapore, increased community transmission resulted in reclosing of borders in December 2021 and January 2022, with the temporary ceasing of VTLs to preserve hospital capacity.^34^ Concomitantly, these policy decisions and the prolonging of PHSMs require an enlarged fiscal space for health.^35^

The availability of technology (indicator 6) is a quintessential element to guide the intensity and duration of PHSMs. This is evident in countries such as Indonesia, Thailand, the Philippines and Vietnam, whereby a large proportion of their student population might not have access to the internet for remote online learning. Despite having measures to support students, it is reportedly insufficient to meet the immense demand. The long-term closure of schools and the digital divide threaten the quality of education and exacerbate inequity gaps.^36^ Access to technology can determine a population’s capacity to maintain physical distancing measures such as WFH arrangements and provision of telehealth services.^37 38^ Furthermore, leveraging digital technologies such as mobile applications to perform check-ins at locations by scanning QR codes and generating proximity notifications when in close contact to a suspected or positive case using Bluetooth technology in addition to alerting individuals and authorities for contact tracing and follow-up management proved vital to reducing the transmission in countries such as Singapore, South Korea and Thailand.^21 39 40^

### PHSMs centred on equity require governance and community engagement to balance the protection of health of population and livelihoods

The phasing introduction of PHSM, either sequentially or concurrently in a comprehensive manner, synergise and complement each other, reflects the decision-making agility of the government based on evidence from the field of what works and vice versa. The up-scaling and down-scaling of PHSMs are mostly guided by epidemiological evidence, the effectiveness of interventions and unintended consequences of policies, reflecting the governments’ adaptability. Therefore, governance structures need to take into account key indicators to derive a plan of action which involves the evaluation of the effects of PHSMs on health of population vs incomes. On another level, strong and coordinated governance is imperative at subnational levels, where local governments are devolved the authority to make decisions while maintaining a coordinated national response at all levels of government.^41^ Instrumentally, governments need to emulate transparency and uphold political accountability regarding decision-making processes, which can come in the form of community engagement as populations need to understand the rationale behind PHSMs, their stringency and duration due to PHSMs’ impacts on their health and livelihoods.^42 43^ Effective and transparent community engagement strategies can help mobilise the general public to action, such as mobilising volunteers to augment the health workforce or to educate and advocate for behavioural changes that promote adherence to PHSMs. This can be achieved through engaging community leaders to promote compliance with physical distancing and mask-wearing measures, leveraging on pre-existing trust fostered within the community.^44^ This is seen in Vietnam, where community COVID-19 supervision groups comprising village health workers, youth or women union volunteers were responsible for sharing information and promoting compliance to PHSMs.^45^ In Singapore, the government also launched both WhatsApp and Telegram chat groups to directly engage the public and provide accurate updates on the evolving situation, modifications to regulations and dispel misinformation.^46^ Notably, there is a need for clear communication channels by the government to clarify vaccine efficacy and safety, and timely management of vaccine misinformation in the Philippines, where vaccine hesitancy was spreading during the initial stages of vaccination roll-out.^47^ Chiefly, community engagement can strengthen governance by providing feedback channels to understand the local sociocultural context, enabling iterative tailoring and cocreation of PHSMs that fit the needs of the population.^48^

All PHSMs seek similar objectives of curbing onward transmission, reducing mortality and preserving livelihoods. The unintended consequences of PHSMs can be seen through widening income gaps, increased gender-based violence and forecasted lowering of educational attainment, among other social and health indices.^49^,^51^ These inequities and inflictions on population health outcomes will not only impact the present but future health of a population and its national economy. Hence, PHSMs need to place equity at the core at all stages of the pandemic and can only be achieved when governance structures put the most vulnerable first while simultaneously balancing the dichotomous trade-offs between the health of population and livelihoods.^52 53^

### Charting an equitable and resilient recovery

As more variants and subvariants such as Omicron BA.1 and BA.2 continue to emerge, the need for contextually appropriate PHSMs that will augment vaccines due to waning of immunity in the population remains.^54^,^56^ Henceforth, governments must remain vigilant and steadfast by executing regular risk assessments through surveillance strategies and reintroducing evidence-based and cost-effective PHSMs.^53^ Although PHSMs serve to safeguard the lives and livelihoods of all populations, including the vulnerable, governments need to redress the existing vulnerabilities and societal inequities. The pandemic has put a wedge on and expanded the current fissures between the have and have-nots and amplified the inequities in all dimensions, not only in LMICs but in high-income countries as well.^57 58^ This is observed in countries reviewed, whereby support packages were offered to vulnerable populations such as migrant workers, as most of them are socially marginalised, lack social protection and are more predisposed to getting infected.^59^ This is also seen in high-income countries such as Singapore, whereby massive outbreaks in crowded foreign worker dormitories led to the implementation of a nationwide Circuit Breaker for 2 months.^60^ Therefore, countries that do not offer vaccines to migrants and non-residents, might observe a persistence of epidemic waves, dampening the effectiveness of other PHSMs. The instance of such oversight is also reported in other vulnerable groups such as internally displaced people and refugees.^61^

Essentially, the level of disproportionate impacts on the poor and vulnerable groups is determined by the level of existing structural inequality on the one hand, the size of social capital and resilience on the other hand, calling on national and global solidarity.^62 63^ In particular, inadequate vaccine coverage and lack of resilient health system pillars can prolong the pandemic as low-income countries continue to fight outbreaks, permitting the emergence of new variants and spread across the world due to international travel.^64^ Increasingly, as more viable and effective antivirals are developed and authorised for use, their equitable access has also come under scrutiny.^65^ Therefore, countries need to focus on equitable distribution of vaccines, antivirals and other resources to strengthen health systems globally and narrow the gap between the global North and South.^66 67^

Lastly, countries must harness this pandemic as an ephemeral window to strengthen health system capacities by investing in three interlocking pillars: (1) capacity to rapidly detect and react to emerging infectious diseases, (2) capacity to absorb infected individuals while maintaining care continuity for routine management of other diseases and (3) foster an enabling climate for the nurturance of healthy lifestyle and behaviours to promote healthier populations resilient towards diseases.^68^ Only through these steps can populations and health systems become more resilient and steadfast in the face of a prolonged pandemic.

### Strengths and limitations

An in-depth analysis of the interlinks and synergies across nine typologies of PHSMs applied by six Asian countries enabled us to rapidly synthesise a framework for PHSMs where key indicators play a significant role in shaping PHSMs as the pandemic unfolds. Importantly, this study confirms a prior study that explores the major negative impacts on social inequity and vulnerability, and that universality principles prevail in the effective containment of the pandemic.^69^ However, this study captures snapshots of policy responses from January 2020 to January 2022 in the context of data incompleteness. Given the ongoing and rapid changes to contextual environments, it is likely that many of the policies highlighted above may have changed considerably since the time of writing. Certain information on policies might also be unavailable and inaccessible from public domains. It is, however, beyond the scope of this study to assess the full range of PHSMs and ascertain the effectiveness of implementation.

## Conclusion

In 2022, as countries are transitioning from acute to recovery phase, where SARS-CoV-2 may become endemic, high levels of vaccine coverage in parallel with the PHSMs explicated in the framework needs to be made permanent features of health system responses. With waning vaccine immunities, newer variant strains and the reopening of economies, health systems need to remain resilient and agile while ensuring that PHSMs can be swiftly reinstated to protect the health of populations and livelihoods with an equity lens.

## Data Availability

Data are available on reasonable request.
